# Utility of metabolic screening in neurological presentations of infancy

**DOI:** 10.1002/acn3.51076

**Published:** 2020-06-04

**Authors:** Djurdja Djordjevic, Etsuko Tsuchiya, Megan Fitzpatrick, Neal Sondheimer, James J. Dowling

**Affiliations:** ^1^ Division of Neurology Hospital for Sick Children Toronto ON Canada; ^2^ Division of Clinical and Metabolic Genetics Hospital for Sick Children Toronto ON Canada; ^3^ Program for Genetics and Genome Biology Hospital for Sick Children Toronto ON Canada; ^4^ Departments of Pediatrics and Molecular Genetics University of Toronto Toronto ON Canada

## Abstract

**Background:**

The first‐line use of specialized metabolic screening laboratories in the investigation of hypotonia and/or developmental delay remains a standard practice despite lack of supporting evidence. Our study aimed to address the utility of such testing by determining the proportion of patients whose diagnosis was directly supported by metabolic screening.

**Methods:**

We performed a retrospective chart review study of 164 patients under age one who had screening metabolic laboratory testing done within the time period of one calendar year.

**Results:**

Of patients screened, 9/164 (5.5%) had diagnoses supported by metabolic testing (two with nonketotic hyperglycinemia, three with ornithine transcarbamylase deficiency, one with propionic acidemia, one with a congenital disorder of glycosylation, one with D‐bifunctional protein deficiency, and one with GM1 Gangliosidosis). Of patients specifically evaluated for hypotonia and/or developmental delay, 5/79 (6.3%) were diagnosed with the aid of metabolic testing. All patients with positive screens presented with acute decompensation. Outside of this subgroup of high‐risk patients, no patients were diagnosed using metabolic testing. Screening laboratories were also ineffective in an outpatient setting, identifying only one of the seven outpatients who was ultimately diagnosed with an inborn error of metabolism.

**Conclusions:**

These findings demonstrate that the yield of specialized metabolic screening testing is extremely low in infants with hypotonia and/or developmental delay, approaching zero outside of the specific setting of clinical decompensation or multi‐system involvement. Furthermore, many outpatient cases of IEM are not identified by screening studies. This information will help guide the diagnostic evaluation of hypotonia and/or global developmental delay.

## Introduction

The underlying conditions resulting in hypotonia and/or developmental delay are numerous, and can be grouped into central nervous system causes (accounting for 60‐80% of cases) and peripheral nervous system causes.^1^ Among central causes, chromosomal abnormalities^2^ and hypoxic ischemic encephalopathy (HIE) represent the most frequent etiologies, accounting for approximately 30% and 20% of neonatal hypotonia cases, respectively,^1^ with other genetic conditions, congenital brain abnormalities, intracranial processes such as hemorrhage, and metabolic disorders accounting for the remainder.^2^

Inborn errors of metabolism (IEM) comprise a rare subset of the conditions associated with hypotonia and/or global developmental delay (GDD).^1^ Previous studies have shown that biochemical testing for IEMs yields a diagnosis in a minority of patients (3% of cases in one series,^1^ 6% in another^2^). In a retrospective review of 144 hypotonic newborns, more than 30% had metabolic investigations, and only 6% contributed to diagnosis; notably, the majority of these infants had multi‐systemic involvement.^3^ Several other publications have suggested that IEMs be considered only in cases of hypotonia and multi organ involvement.^1^, ^4^, ^5^, ^6^ The yield is also low (1‐5% in most studies^7^, ^8^) in patients with isolated GDD or intellectual disability. Despite these largely negative data, metabolic screening remains a recommended first‐line test.

Screening metabolic laboratory tests typically include plasma amino acids, urine organic acids, acylcarnitine profile, ammonium, and may extend to urine oligosaccharides, urine mucopolysaccharides, and studies of protein glycosylation.^9^, ^10^, ^11^ With the widespread employment of tandem mass spectrometry‐based newborn screening, the previously quoted numbers in terms of the yield of metabolic testing are likely to be even lower, as progressively greater numbers of IEM are identified by NBS at the time of birth. In contrast, several recent studies have shown that Whole‐Exome Sequencing provides diagnostic resolution in over 40% of patients with developmental delay.^12^

Due to this changing landscape, the recommendations for the diagnostic evaluation of infants with hypotonia and/or GDD remain unclear. The Canadian Pediatrics Society position statement recommends a subset of metabolic screening laboratories as first‐line investigations alongside chromosomal microarray, fragile X and brain MRI.^8^ Despite their low yield, the position paper argues in favor of including metabolic screening as a first‐tier test because of the treatability of many IEMs.^8^ Conversely, the American Academy of Pediatrics recommends against global screening, and rather recommends use of these investigations when there is clinical suspicion for an IEM.^13^ Despite this, it is still standard practice in US academic institutions to include metabolic tests when evaluating infants with hypotonia or GDD.

The true utility of metabolic testing in hypotonia and/or GDD remains to be adequately studied. Our primary objective is to determine the diagnostic yield of screening metabolic tests in this setting. Secondarily, the study aimed to identify the subset of infants, if any, who are likely to benefit from these investigations, and to better understand the current state of testing and diagnosis for neurological presentations in the neonatal population.

## Methods

A retrospective chart review was performed on all patients under 1 year of age within the year 2017 who had specialized metabolic laboratory testing done at the Hospital for Sick Children (HSC). All metabolic testing at HSC, including inpatients and outpatients, is performed at a single diagnostic laboratory. Institutional Research Ethics Board (REB) approval was obtained prior to initiation of the study and included a waiver of informed consent. This study was conducted in accordance with: The Government of Canada’s Tri‐Council Policy Statement: Ethical Conduct for Research Involving Humans (TCPS2); ICH Harmonized Tripartite Guideline: Guideline for Good Clinical Practise E6 (ICH GCP E6); and The Declaration of Helsinki.

Subjects were ascertained based on having had serum amino acid and/or urine organic acid testing. While metabolic screening consists of a broader set of laboratories,^10^, ^11^ we considered that these were the most discriminatory in terms of identifying subjects who underwent specialized metabolic testing. Of the 456 patients meeting the initial criteria (i.e., having had amino acid or organic acid studies), chart review identified 324 patients who had these tests done for diagnostic purposes. A total of 164/324 (51%) of these were done for neurologically related presentations. Laboratory testing was reviewed to determine which metabolic screening tests were ordered; specialized screening laboratories were considered to include plasma amino acids, urine organic acids, acylcarnitine profile, urine oligosaccharides, urine mucopolysaccharides, plasma very long chain fatty acids (VLCFA), transferrin glycosylation, and ammonium. Abnormal results suggestive of an IEM were considered positive screening tests. Lactate and glucose were not considered screening tests because of their extensive and general use within the hospital and their lack of specificity for IEMs.

The primary objective of this study was to quantify the number of patients presenting with hypotonia and/or developmental delay who had positive metabolic screening laboratories leading to diagnosis, in order to ascertain the yield of these investigations. Secondarily, the study aimed to identify qualitatively the characteristics of these patients, in order to identify subsets of patients in whom these investigations may be of higher yield. Post hoc qualitative analysis was further done to examine the underlying etiologies and method of diagnosis of patients presenting with hypotonia/GDD, as well as seizures as this also represented a large subset of the patients. Further description can be found in the supplemental methods.

## Results

### All patients

**Figure 1:**
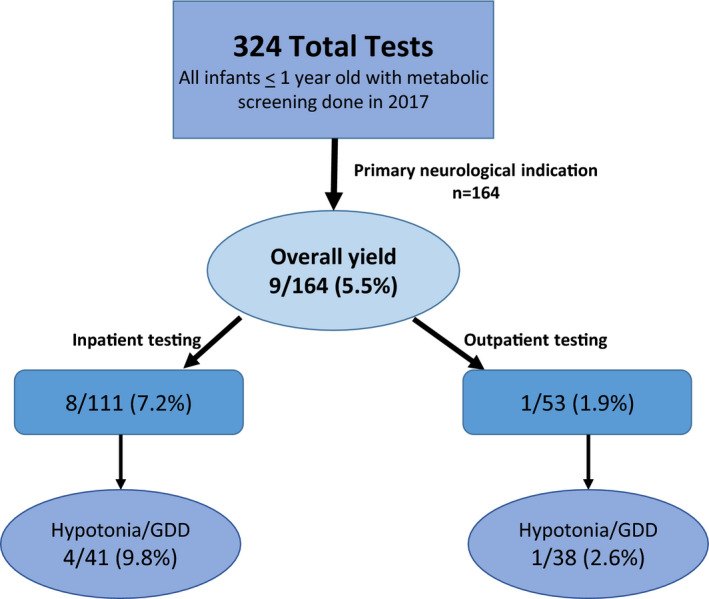
Metabolic screening for neurologic indications in infants. Flow chart of the data presented in the manuscript. This retrospective study included all cases of infants <1 year of age in 2017 who had metabolic screening laboratory studies performed at Hospital for Sick Children in Toronto. 324 unique cases were represented in this cohort. A total of 164 screens were performed where the primary test indication was neurologic, including hypotonia, global developmental delay, seizures, movement disorder, and stroke. In 9/164 cases overall (5.5%), metabolic screening was positive and guided diagnosis. Cases were further subdivided by whether they were sent from the inpatient (111) or outpatient (53) setting, and for the specific indication of hypotonia and/or global developmental delay. In all settings, the percentage of positive tests was extremely low. (yield = percentage of positive screens/total patients where screening metabolic studies were performed).

A total of 164 infants (<1 year old) had specialized metabolic screening laboratories done at the Hospital for Sick Children (Toronto, Canada) in 2017 for neurologically related presentations. Nine patients had diagnoses that were aided by these tests (9/164 = 5.5%, Figure 1). Diagnoses included nonketotic hyperclycinemia (NKH) (n = 2), ornithine transcarbamylase deficiency (OTC) (n = 3), propionic academia (n = 1), congenital disorder of glycosylation 1a (CDG1a) (n = 1), D‐bifunctional protein deficiency (DBP) (n = 1), and GM1 Gangliosidosis (n = 1). Additional details for each positive screen case are presented in Table 1.

**Table 1:** Case details for positive metabolic screens (n = 9/164).

Positive test	Clinical features	Other laboratory results	Imaging	Diagnosis
Plasma AA (glycine elevated), then CSF AA and urine AA showing high glycine	Progressive encephalopathy, seizures	urine organic acids, ACP, carnitine, CSF lactate/glucose, CDG, TSH, CPK, VLCFA (all WNL)	MRI normal	NKH
Plasma AA (glycine elevated), then CSF AA and urine AA showing high glycine	Congenital hypotonia, Seizures, encephalopathy	Urine organic acids, ACP, carnitine, TSH, muco/oligosach (all WNL)	Not done	NKH
Ammonium, then acylcarnitines (ACP) and urine organic acids	Respiratory distress, difficulty feeding		HUS normal before diagnosis	Propionic acidemia
Ammonium, then plasma amino acids	Dyspnea, encephalopathy, hypotonia, seizures	Urine organic acids (WNL)	MRI brain normal after diagnosis	Ornithine Transcarbamylase Deficiency
CDG transferrin	FTT, GDD, hypotonia, admitted for respiratory decompensation/ septic picture	Plasma amino acids, TSH (all WNL)	Not done	CDG1a
Ammonium, then plasma amino acids	Seizures, lethargy	urine organic acids (WNL)	MRI done before diagnosis normal	Ornithine Transcarbamylase Deficiency
Ammonium, then plasma amino acids	Progressive encephalopathy and seizures unresponsive to abortive meds	ACP, carnitine, CPK (all WNL)	Not done	Ornithine Transcarbamylase Deficiency
MRI brain, then VLCFA	Congenital hypotonia, hypoglycemia	serum amino acids, urine organic acids, ACP, carnitines, CPK (all WNL)	MRI brain—polymicrogyria bilateral frontoparietal lobes, multiple subependymal cysts, pons small	D‐bifunctional protein deficiency (WES positive for *HSD17B4* homozygous mutation)
Urine oligosaccharides	GDD, severe hypotonia, coarse features, hepatomegaly	Serum amino acids, urine organic acids, ACP, carnitines, urine MPS, TSH, ammonia (all WNL). Beta gal activity abn (WBCs)	MRI brain normal	GM1 gangliosidosis

AA, amino acids; ACP, acylcarnitine profile; CDG, congenital disorders of glycosylation; CPK, creatine phosphokinase; CSF, cerebral spinal fluid; TSH, thyroid stimulating hormone; VLCFA, very long chain fatty acids; WNL, within normal limits.

Of the 164 cases, 79 screens were done for a primary indication of hypotonia and/or developmental delay (69 patients with hypotonia with or without GDD, and 10 additional patients evaluated for GDD without the presence of hypotonia). In this group, a total of 5/79 (6.3%) had their diagnosis supported by metabolic screening tests (Tables 2 and 3).

**Table 2:** Yield of each investigation for the diagnosis of hypotonia/GDD, in the inpatient and outpatient settings, according to number of patients who had each investigation completed.

Investigation (number tested)	Total (n = 79)	Inpatients: (n = 41)	Outpatients: (n = 38)
Metabolic screening	5/79 (6.3%)	4/41 (9.8%)	1/38 (3%)
WES	23/28 (82.1%)	11/15 (73%)	12/13 (92%)
Microarray	14/71 (19.7%)	6/33 (18%)	8/38 (21%)
MRI	9/72 (12.5%)	7/37 (19%)	2/35(6%)
Multi gene panel	4/6 (66.7%)	1/2 (50%)	3/3 (100%)
Single‐gene test	1/1 (100%)	0/0	1/1 (100%)
Results pending/ no other tests sent	18/79 (22.8%)	8/41 (19%)	10/38 (26%)

**Table 3:** Underlying diagnoses for patients with hypotonia/developmental delay who had metabolic screening tests either in the inpatient or outpatient setting.

Diagnosis	Total (n = 79)	Inpatient: (n = 41)	Outpatient: (n = 38)
Prader WIlli	5 (6.3%)	5 (12%)	0
HIE	5 (6.3%)	5 (12%)	0
Other neuro malformation/insult (cortical, bleed, infection, hypoxia)	2 (2.5%)	1 (2%)	1 (3%)
IEM	11 (13.9%)	4 (10%)	7 (18%)
Single‐gene mutation (nonmetabolic/ mitochondrial/ neuromuscular)	24 (30.4%)	10 (24%)	14 (37%)
Deletion/duplication/ chromosomal	8 (10.1%)	1 (2%)	7 (18%)
Neuromuscular/ mitochondrial	9 (11.4%)	6 (15%)	3 (8%)
No diagnosis: negative investigations including WES	4 (5.0%)	3 (7%)	1 (3%)
No diagnosis: incomplete testing	11 (13.9%)	6 (14%)	5 (13%)

### Inpatient setting

There were 111 inpatients screened with metabolic testing. Of the 111 patients, nine had a confirmed diagnosis of an IEM (9/111 = 8.1%). Of these, eight were initially suspected via metabolic screening laboratories (8/111 = 7.2%). All eight were acutely unwell, presenting with acute decompensation (acute encephalopathy, sepsis‐like picture, and/or seizures). Two were diagnosed with NKH, three were diagnosed with OTC, one with propionic academia, one with DBP, and one was diagnosed with CDG1a. Of the inpatients diagnosed by metabolic screening, all screens were completed within 1‐2 days of the investigations being sent. Notably, chart review of the patients with treatable conditions (OTC and PA) shows that therapies were initiated based on presentation and prior to the return of metabolic testing results, confirming that these treatable IEMs are frequently suspected and diagnosed by clinical picture, with laboratory studies providing secondary confirmation.

Of the 111 inpatients, 41 were for a primary indication of hypotonia/GDD, with four testing positive to suggest a primary metabolic disease (4/41 = 9.8%) (one NKH, one OTC, one DBP, one CDG1a). Outside of these four cases, there were no diagnoses of hypotonic/ developmentally delayed patients made by metabolic screening. WES was the highest yield investigation, diagnosing 11/15 where the test was sent, with an overall yield of 73% (Tables 2 and 3).

### Outpatient setting

A total of 53 outpatients had screening metabolic studies. In these patients, 28 had hypotonia with or without GDD, and an additional 10 were screened due to developmental delay or regression in the absence of hypotonia on examination. Of these 38 patients, only one patient (with GM1 gangliosidosis) was identified via metabolic screening (1/38 = 2.6%). Clinically, this patient was profoundly hypotonic and developmentally delayed, with findings including dysmorphic features and organomegaly on examination, and a high prescreening suspicion for a storage disorder.

All 38 patients with GDD and/or hypotonia had chromosomal microarray, and 8/38 (21%) were diagnosed with this methodology, in keeping with previous reports. WES was the highest yield investigation in this cohort, diagnosing 12/13 patients with GDD/hypotonia who had testing (Tables 2 and 3). Notably, all 13 patients had metabolic screening and microarray sent before WES. Overall, 61% of patients were ultimately diagnosed with single gene or chromosomal abnormalities by microarray, multi gene panel, or WES. MRI brain suggested a specific diagnosis in one patient with developmental regression (Krabbe disease), and was abnormal in a total of 16/38 patients; the majority of these (9/16) had imaging completed prior to genetic testing.

Seven of 53 outpatients screened with specialized metabolic laboratories for neurological indications ultimately had a diagnosis of an IEM. Of note, only 1/7 was identified by specialized metabolic testing (positive urine oligosaccharides in a patient with GM1 gangliosidosis). The other six patients with IEM were diagnosed via WES, microarray, or targeted gene testing, and had negative metabolic screening laboratories (case details in Table 4). Two of seven patients had episodes of hypoglycemia, with one diagnosis subsequently established via multigene panel (glycogen storage disease 3A) and one via WES (glycogen storage disease 9A).

**Table 4:** Details of IEM cases with negative specialized metabolic testing.

Diagnosis	Specialized metabolic testing results	Clinical features	Imaging	Method of Diagnosis
Lipoic acid synthase deficiency	Urine OA, ammonium, carnitines = normal. Plasma AA initially elevated glycine but normalized on repeat testing. Urine sulfites, normal. No TSH, VLCFA.	Refractory neonatal seizures, imaging consistent with HIE but history not in keeping	HUS ‐ symmetric increased WM echogenicity, cystic changes in caudothalamic grooves; MRI‐diffuse WM signal abnormalities and diffusion restriction	WES (expedited due to acuity): lipoid acid synthase deficiency
Smith Lemli Opitz syndrome	Urine OA, ACP, carnitines, serum AA, homocysteine, MPS, CDG, biotinidase, folate, ammonia = normal	Hypotonia, feeding difficulties, GDD, dysmorphic features	MRI‐ low brain volume, mild delay in myelination	WES (two pathogenic mutations in *DHCR7* gene)
Glycogen storage disease 3a	Urine OA normal; carnitines, ACP, serum AA, urine AA normal	Recurrent episodes of ketotic hypoglycemia, hepatomegaly, encephalopathy/ irritability/ inconsolability	None	multi gene panel (homozygous deletion in *AGL* gene)
Phosphoglycerate dehydrogenase deficiency	Serum AA, urine OA = normal	Significant microcephaly, increased appendicular tone, mild dysmorphic features	MRI simplified gyro pattern on MRI with delayed myelination, thin CC	WES (homozygous pathogenic mutation in *PHGDH* gene)
Glycogen storage disease 9	Urine OA, carnitines, ACP, quant AA, ammonium = normal	Ketotic Hyoglycemic episodes, poor growth, GDD	None	Targeted testing (sibling diagnosed by WES) (mutation in *PHKA2* gene)
Lesch‐Nyhan syndrome	Plasma AA, urine OA, MPS, CK normal	Profound axial hypotonia, GDD. Consanguineous parents	MRI showed prominence of CSF spaces suggestive of EVOH	WES (*HPRT1* pathogenic variant)
Krabbe disease	Ammonium, CDG, carnitines, pyruvate, plasma AA, urine OA, ACP, creatine disorders panel, urine oligosacch, urine MPS, urine AA = normal	Developmental regression (severe), abnormal posturing + hypertonia	MRI brain suggestive of leukodystrophy in keeping with Krabbe	Galactocerebrosidase activity low (0.6). Genetic testing for *GALC* gene (pathogenic homozygous variant)

AA, amino acids; ACP, acylcarnitine profile; CC, corpus callosum; CDG, congenital disorder of glycosylation; EVOH, *ex vacuo* hydrocephalus; MPS, mucopolysacchridoses; OA, organic acids; TSH, thyroid stimulating hormone; VLCFA, very long chain fatty acids.

### Other indications

A total of 66 patients were evaluated where seizures was their primary indication. Of these, 3/66 (4.5%) had positive metabolic screening: one with NKH (positive screen with plasma amino acids) and two with OTC (positive screens with ammonium). All three patients evidenced acute decompensation with intractable seizures. Otherwise, patients evaluated for seizures were most frequently diagnosed via brain MRI (54%), with HIE or cortical malformation being the most frequently encountered diagnoses. Epilepsy gene panel, metabolic screening laboratories, microarray, and WES, respectively, were the next most common methods of diagnosis (accounting for roughly 20%) (Table 5).

**Table 5:** Diagnostic yield by investigation for cases where the primary indication listed for metabolic testing was seizures.

Investigation	Patients presenting with seizures (n = 66)
WES	2 (3%)
Microarray	2 (3%)
MRI	36 (54%)
Metabolic screening	3 (5%)
Genetic panel	5 (8%)
No diagnosis/other	18 (27%)

No patients with stroke/intracranial bleed (4), movement disorder (2), abnormal eye movements (2), or brain malformations (11) were diagnosed by metabolic screening laboratories. The yield of investigations for all neurological presentations combined is presented in Table 6.

**Table 6:** Percentages of all patients with neurological presentations diagnosed using each investigation, in the inpatient and outpatient setting.

Investigation	Outpatients: (n = 53)	Inpatients: (n = 111)
WES	13 (24%)	12 (11%)
Microarray	8 (15%)	10 (9%)
MRI	2 (4%)	39 (35%)
Metabolic screening	1 (2%)	8 (7%)
Genetic panel	3 (7%)	6 (5%)
Targeted gene	1 (2%)	1 (1%)
Negative WES	1 (2%)	4 (4%)
Pending results or no other testing sent	24 (45%)	31 (28%)

## Discussion

IEMs are rare and potentially treatable etiologies for neurological presentations in the first year of life. IEMs are also on the differential diagnosis as causes of global developmental delay and/or hypotonia. Because of this, a group of specialized screening metabolic laboratories is traditionally recommended as a first‐line test for these indications. We addressed the yield of this approach in an unbiased cohort (i.e., based only on having had testing in the first year of life) from a single center for an entire calendar year. We found an extremely low yield for metabolic screening. Our key finding is that metabolic testing was unhelpful outside of cases presenting with acute neurologic decompensation, and a single case of encephalopathy with dysmorphic features and organomegaly. Therefore, based on our data, outside of these specific contexts, we propose that specialized metabolic screening should not be considered as a first‐line test in patients presenting with hypotonia and/or global developmental delay.

Of all patients evaluated for hypotonia and/or developmental delay, 6.3% were diagnosed with the aid of specialized metabolic screening laboratories, which is similar to the yield found in previous studies (between 1 and 5% for all cases of global developmental delay in most studies,^8^ 3‐6% of all cases of neonatal hypotonia^1^, ^2^). This is in contrast to the yields reported in the literature for microarray (approximately 10‐20%) and WES (approximately 40‐50%^12^). In our cohort, the yield of microarray was 21% of all outpatients and 18% of inpatients. Most strikingly, the yield of WES was extremely high in this study. Within the outpatient group, 32% of the overall cohort of hypotonic/delayed outpatients received a diagnosis using WES, with a diagnostic yield of 92% for the patients who actually were tested. The yield was also high in the inpatient group, where 73% of patients who were tested by WES received a diagnosis. All of these patients had metabolic screening and microarray sent before WES, resulting in delay of utilization of the highest yield test. Indeed, WES outperformed metabolic screening even for the identification of IEMs. Notably, of seven patients ultimately diagnosed with an IEM who had metabolic screening as outpatients, only one of seven patients was identified by this approach. This suggests that not only are metabolic laboratory tests of very low yield, but they are not adequate to identify patients with IEM, particularly in the outpatient setting. Of note, at present the approximate cost (in CAD) of specialized metabolic laboratory tests (e.g., serum amino acids, urine organic acids, VLCFA, and acylcarnitine profile) is approximately $1000, and the current cost of trio‐based whole‐exome sequencing is approximately $3000 (with price continuing to decrease).

In this study, the independent examination of inpatients and outpatients allowed for the identification of a patient group that was likeliest to benefit from metabolic screening. Essentially all of the inpatients with positive screening laboratories were acutely unwell, manifesting encephalopathy, progressive and intractable seizures, and/or neurological deterioration. Metabolic testing is valuable and beneficial in this setting, particularly given that rapidly establishing a diagnosis of an IEM may lead directly to therapeutic intervention. This is particularly true for infants who are less than 1 week old, for whom mandated newborn screening would not have yet been completed.

Conversely, the outpatient setting comprises a group of patients with hypotonia/GDD with a more chronic or indolent course. The majority of these patients have completed newborn screening, which substantially decreases the number of patients who are likely to be diagnosed with IEM in the outpatient setting. Importantly, the diagnostic guidelines as to the optimal investigation of developmentally delayed or hypotonic patients refer more to this patient population, rather than to those presenting with acute deterioration. In this group, metabolic screening laboratories were extremely low yield, with only a single patient (1.9%) being diagnosed via these tests in our cohort. This specific patient had a clinical course and exam that was already suggestive of the diagnosis, GM1 gangliosidosis. Furthermore, several cases of IEM in our outpatient cohort were identified by genetic testing and missed by the metabolic screen.

Of note, previous studies have shown that the majority of patients diagnosed with IEM had multi‐systemic involvement.^1^, ^4^, ^5^, ^6^ The Canadian Pediatric Society (CPS) statement does argue that there are patients not diagnosed by newborn screening without findings on physical examination for IEM, who may have an identifiable and treatable condition. Our data suggest that while this may be true, such patients do not appear to present with hypotonia and GDD when multi‐system disease is otherwise not present, and/or do not have positive screening laboratories even when the ultimate diagnosis is an IEM. In keeping with our results, the American Academy of Pediatrics holds the position that metabolic screening laboratories be reserved for patients with GDD in whom there is specific clinical suspicion for an IEM.^13^ Furthermore, we now know that genetic‐based diagnostic studies such as WES allow for the identification of causal mutations in over 40% of patients with developmental delay^12^ suggesting that these studies should not be delayed in lieu of screening laboratory tests. These results also add evidence that genetic‐based testing should be pursued even in the presence of normal initial metabolic screening.

The etiologies underlying cases of hypotonia and/or developmental delay in this study also add to the existing literature of the diagnoses that should be considered. The inpatient and outpatient settings both significantly favored single gene and chromosomal abnormalities, which is in keeping with the current literature. The relatively high rate of IEM diagnoses identified in our study compared to those seen in the literature may be explained by the setting being examined in this study, given it is a referral‐based center where children are seen by metabolics and neurology subspecialists. Notably, despite higher rates of diagnoses of IEM, the yield of metabolic screening tests in their diagnoses remained low. This further supports the recommendation that WES should be considered as the first‐line diagnostic test.

Although previous studies have suggested that HIE is one of the most frequently encountered diagnoses explaining neonatal hypotonia,^1^, ^2^ none of the outpatients in our cohort had brain imaging demonstrating hypoxic ischemic injury, showing the significant skew of this diagnosis toward inpatients, and also potentially reflecting the specialized neonatal neurologic care pathway at our institution. Overall, MRI brain was a low‐yield test in the diagnosis of hypotonia/GDD. Further study is necessary to establish whether MRI should be a first‐line study in patients with GDD and/or hypotonia, or instead should be employed after genetic testing to either add clarification to inconclusive testing results or diagnostic support when WES is negative.

Although defining the yield of metabolic testing in other neurologic conditions presenting in infancy (movement disorders, epilepsy, stroke, etc) was not our primary focus, there are some conclusions that can be drawn from our data. As with developmental delay and hypotonia, the yield was extremely low for these conditions, with no positive tests for movement disorders for example, and only 4/66 (6.1%) for seizures, and only in the setting of acute clinical deterioration. This is in keeping with previous studies looking at these phenotypes in older children. For example, a study of diagnostic utility in a cohort of pediatric movement disorder patients revealed 0/51 cases with abnormal metabolic tests.^14^ Overall, this supports the lack of utility of metabolic testing for neurogenetic disease outside of the specific contexts of acute decompensation or the rare patient with an overwhelmingly suggestive picture (multi organ involvement with dysmorphic features and/or organomegaly).

While this study provides important evidence regarding diagnostic assessment of infantile hypotonia and developmental delay, there are some limitations to note. Foremost is that we did not examine utility of metabolic testing in a prospective manner. Second, we used data from a single center. While it is a quaternary care center that draws patients of different ethnicities and backgrounds from around the world, there is the potential that the results may reflect mainly on the population most highly served by the hospital. Lastly, while sent in many of the cases in our cohort, our study did not systematically evaluate the performance of the entire set of recommended metabolic studies (e.g. lactate, TSH, ALT, AST, CK, glucose, and complete blood counts^10^, ^11^, ^15^), but instead focused on the yield of studies that utilize specialized metabolic laboratories, as these require unique equipment and expertise and are the primary cost drivers of metabolic screening. For example, the two patients with glycogen storage disease (see Table 4), while having normal specialized metabolic testing, had abnormal blood glucose values. We endorse the utility of blood glucose testing in hypotonic infants or other similar low‐cost screenings performed in general chemistry laboratories.

## Conclusion

The results of this study indicate that specialized metabolic screening laboratory tests are low‐yield investigations for the diagnosis of hypotonia and/or global developmental delay, and should be considered second‐line testing, except in specific suggestive circumstances such as acute neurologic decompensation or multi‐systemic involvement suggestive of a specific metabolic condition.

## Conflict of Interest

None of the authors have conflicts of interest that are relevant to the manuscript and data contained therein.

## Author Contributions

DD helped with all aspects of the study. She was primarily responsible for data collection, participated in data interpretation, and generated the first drafts of the manuscript. HG assisted in data collection and interpretation. ET spearheaded all aspects of protocol generation, REB submission, and REB approval. MG assisted in all aspects of REB submissions and approval. NS helped to formulate the study, participated in data interpretation, and edited the manuscript. JJD oversaw all aspects of the study, participated in data interpretation, and helped generate and finalize the manuscript.
